# Genome-wide MicroRNA Expression Profiles in COPD: Early Predictors for Cancer Development

**DOI:** 10.1016/j.gpb.2018.06.001

**Published:** 2018-07-05

**Authors:** Andreas Keller, Tobias Fehlmann, Nicole Ludwig, Mustafa Kahraman, Thomas Laufer, Christina Backes, Claus Vogelmeier, Caroline Diener, Frank Biertz, Christian Herr, Rudolf A. Jörres, Hans-Peter Lenhof, Eckart Meese, Robert Bals, for the COSYCONET Study Group

**Affiliations:** 1 Chair for Clinical Bioinformatics, Saarland University, 66123 Saarbrücken, Germany; 2 Department of Human Genetics, Saarland University Hospital, 66421 Homburg, Germany; 3 Hummingbird Diagnostics GmbH, 69120 Heidelberg, Germany; 4 Department of Internal Medicine, Division for Pulmonary Diseases, Philipps University of Marburg, 35043 Marburg, Germany; 5 Institute for Biostatistics, Hannover Medical School, 30625 Hanover, Germany; 6 Department of Internal Medicine V – Pulmonology, Allergology, Intensive Care Medicine, Saarland University Hospital, 66421 Homburg, Germany; 7 Institute and Outpatient Clinic for Occupational, Social and Environmental Medicine, Comprehensive Pneumology Center Munich (CPC-M), Ludwig-Maximilians-University Munich, Member of the German Center for Lung Research (DZL), 80539 Munich, Germany; 8 Chair for Bioinformatics, Saarland University, 66123 Saarbrücken, Germany; 9 Center for Bioinformatics, Saarland University, 66123 Saarbrücken, Germany

**Keywords:** microRNA, Cancer, COPD, Lung, Biomarker, COSYCONET

## Abstract

Chronic obstructive pulmonary disease (**COPD**) significantly increases the risk of developing **cancer**. **Biomarker** studies frequently follow a case-control set-up in which patients diagnosed with a disease are compared to controls. Longitudinal cohort studies such as the COPD-centered German COPD and SYstemic consequences-COmorbidities NETwork (**COSYCONET**) study provide the patient and biomaterial base for discovering predictive molecular markers. We asked whether **microRNA** (miRNA) profiles in blood collected from COPD patients prior to a tumor diagnosis could support an early diagnosis of tumor development independent of the tumor type. From 2741 participants of COSYCONET diagnosed with COPD, we selected 534 individuals including 33 patients who developed cancer during the follow-up period of 54 months and 501 patients who did not develop cancer, but had similar age, gender and smoking history. Genome-wide miRNA profiles were generated and evaluated using machine learning techniques. For patients developing cancer we identified nine miRNAs with significantly decreased abundance (two-tailed unpaired *t*-test adjusted for multiple testing *P* < 0.05), including members of the miR-320 family. The identified miRNAs regulate different cancer-related pathways including the MAPK pathway (*P* = 2.3 × 10^−5^). We also observed the impact of confounding factors on the generated miRNA profiles, underlining the value of our matched analysis. For selected miRNAs, qRT-PCR analysis was applied to validate the results. In conclusion, we identified several miRNAs in blood of COPD patients, which could serve as candidates for biomarkers to help identify COPD patients at risk of developing cancer.

## Introduction

Chronic obstructive pulmonary disease (COPD) is a common disease that affects several hundred million people worldwide. It represents one of the most common causes of death. According to the World Health Organization, in 2015, COPD is estimated to have caused the death of 3.17 million patients (http://who.int/mediacentre/factsheets/fs310/en/). COPD is considered a systemic disease and is often associated with comorbidities that have significant impact on the quality of life [1]. Patients suffering from COPD have also an increased risk of cancer [2]. In particular, COPD increases the risk of developing lung cancer significantly [3].

The underlying mechanisms of the increased cancer risk in COPD patients are unclear but probably involve the presence of chronic inflammation, increased levels of oxidative stress leading to DNA damage, exposure to cytokines that repress the DNA repair mechanisms [3], and the changes of the pulmonary microbiota [4]. Specifically, the activation of NF-κB plays an important role in the development of lung cancer from COPD [5]. In addition, COPD patients have an increased risk of a worse outcome after lung cancer diagnosis and treatment [6]. In sum, there is growing evidence suggesting a close association between persistent chronic inflammation and lung cancer [7] and even evidence for a causal relationship between inflammation and lung cancer [8]. Consequently, early diagnosis of lung cancer is of exceptional importance for individuals affected by COPD. Hence, there is a clinical need for biomarkers that indicate an increased risk of cancer development in COPD patients.

Lung cancer is, however, not the only cancer comorbidity in COPD patients. Several cohort studies revealed an increased risk of other, mostly tobacco-related cancers. A nationwide Danish study shows increased incidences for cancers of the lung, larynx, tongue, oral cavity, pharynx, esophagus, stomach, liver, pancreas, cervix uteri, and urinary tract in COPD patients, with standardized incidence ratios between 1.3-fold and 2.8-fold [9]. Similar results have also been reported in a nationwide cohort study in Taiwan showing a 2.8-fold increase in hazard ratio of COPD patients for developing cancers as compared to controls without COPD after adjusting for age, sex and comorbidities [2]. The most common cancers reported in this study are lung, liver, colorectal, breast, prostate, and stomach cancers [9].

Besides the consumption of tobacco as the most common cause of COPD, age is an important risk factor for COPD [10]. Moreover, a number of single nucleotide variants (SNVs) in the genome are known to be associated with COPD [11], [12], [13]. Besides single nucleotide polymorphisms (SNPs) in protein-coding genes, SNPs in RNAs that are not transcribed seem to play a role in the development of COPD as well [14], [15]. One important class, belonging to the small non-coding RNAs (sncRNAs), comprises microRNAs (miRNAs) that are 21–23 nucleotides in length. Several studies have reported tissue-based miRNA profiles or circulating miRNA profiles in COPD [16], [17]. Studies aiming at biomarker discovery and/or validation provide the first evidence for miRNAs as potential markers for lung cancer [18], [19], [20] and other tumor types [21]. miRNAs have also been discussed as regulators of airborne pollution-induced lung inflammation and carcinogenesis [22]. Some miRNAs are not linked to one single cancer type, but may be dysregulated in multiple cancer types or even in diseases in general, such as miR-144 [23]. A substantial fraction of miRNA lung biomarker studies rely on case–control set-ups where diseased patients are compared with a control cohort of unaffected individuals or with patients suffering from diseases with similar symptoms [24].

Being aware of both the promises and challenges of miRNAs for a future diagnostic applications in general [25] and for lung cancer specifically [26], we asked whether blood-based miRNA signatures of COPD patients who develop cancer within a given time window, are different from COPD patients who do not develop cancer. To answer this question, a large set of COPD patients who have been followed up for several years is required for the identification of cancer-predictive miRNA profiles. The German COPD and SYstemic consequences-COmorbidities NETwork (COSYCONET) study has collected biomaterial and clinical information of COPD patients with a follow-up time of 54 months [27]. The study set-up allows us to compare molecular baseline measurements with clinical parameters. We are also able to investigate whether the COPD patients who did not have cancer diagnosis at baseline, but with cancer developed over time, show molecular patterns associated with the development of cancer.

## Results

**Figure 1:**
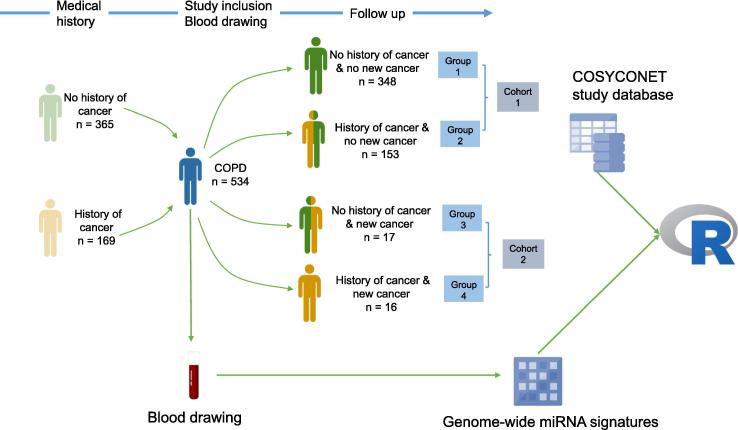
**Experimental design** We profiled individuals with COPD (blue) who either did not develop cancer (Cohort 1) or developed cancer (Cohort 2) during the 54-month follow-up. Genome-wide blood-borne miRNA profiles were generated for all 534 patients. These expression data were correlated with clinical data from the COSYCONET data repository using statistical approaches implemented in the programming environment R. COPD, chronic obstructive pulmonary disease; COSYCONET, COPD and SYstemic consequences-COmorbidities NETwork.

The study set-up is sketched in Figure 1. We analyzed genome-wide miRNA profiles for 2549 mature human miRNAs of blood samples collected from 534 COPD patients participating in the COSYCONET study. Details on the patient cohorts including age, gender, and smoking status are provided in Table 1.

**Table 1:** **COPD patient characteristics**

	**Cohort 1**	**Cohort 2**	**Statistical significance**
Gender (female/male)	179/322	8/25	n.s.
Smoker (never/past/present)	21/358/122	1/28/4	n.s.
Age (mean ± sd)	67 ± 7.8	69 ± 5.9	n.s.
Packyears (mean ± sd)	47 ± 36.7	61 ± 37.8	n.s.

*Note*: n.s., not significant.

### Nine significant miRNAs predict the development of cancer

Supplementary Tables S1–S5 associated with this article can be found, in the online version, at https://doi.org/10.1016/j.gpb.2018.06.001.

**Figure 2:**
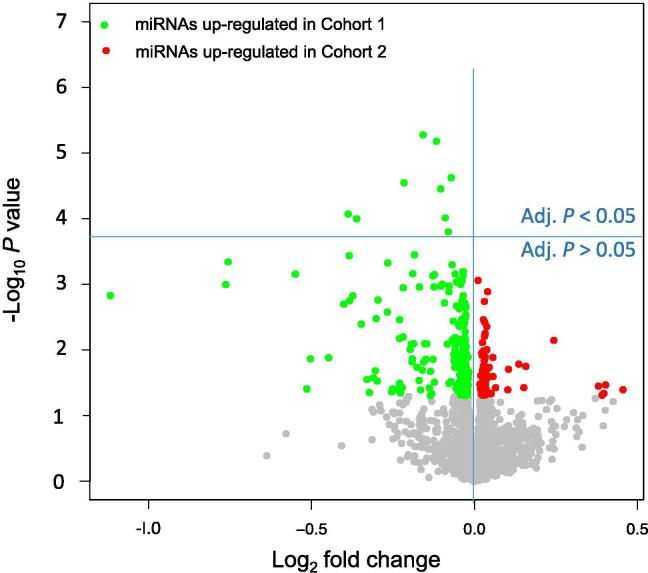
**Volcano plots for the pair-wise comparison of patients developing cancer and patients not developing cancer** Volcano plots show on the *Y*-axis the negative log_10_*P* value of the *t*-test and on the *Y*-axis the log_2_ fold change. Green dots indicate miRNAs with higher expression in blood samples from Cohort 1, while red dots represent miRNAs with higher expression in blood samples from Cohort 2 according to the raw *P* < 0.05. All dots with raw *P* ≥ 0.05 are colored in gray. The horizontal blue line separates miRNAs that are significant following Benjamini–Hochberg adjustment (above the line; adjusted *P* < 0.05) and miRNAs that are not significant anymore following the adjustment (below the line).

We compared miRNA expression profiles in patients not developing cancer (n = 501; Cohort 1 in Table 1) to those developing cancer (n = 33; Cohort 2 in Table 1) within a follow-up period of up to 54 months. Out of the 2549 profiled miRNAs, 269 (10.6%) were nominally significant (*i.e.*, unadjusted *P* < 0.05; *t*-test), including 199 showing decreased expression and 70 showing increased expression in patients developing cancer. Although the number of features, *i.e.*, miRNAs in this study (*P* = 2549), as measured in the study was substantially above the total cohort size (n = 534), nine miRNAs still remained statistically significant after adjustment for multiple testing using the Benjamini–Hochberg approach. These include hsa-miR-548d-5p, hsa-miR-4695-3p, hsa-miR-517a-3p, hsa-miR-4785, hsa-miR-7109-3p, hsa-miR-320e, hsa-miR-548ay-5p, hsa-miR-320c, and hsa-miR-519d-3p. In accordance with the observation that the expression of most miRNAs in patients developing cancer was reduced, all of these nine miRNAs showed a decreased abundance as well. An overview of the miRNA expression is presented as Volcano plot in Figure 2, showing the negative log_10_ *P* value versus the log_2_-fold change of the miRNA. Besides the parametric *t*-test, we also applied the non-parametric Wilcoxon–Mann–Whitney test. Both tests showed a high concordance (Pearson correlation of 0.76, *P* < 10^−16^), although none of the miRNAs remained significant following adjustment for multiple testing in this rank-based test. A complete list of raw and adjusted *P* values for both hypothesis tests is provided for all 2549 miRNAs in Table S1, together with the median expression of each miRNA in the two cohorts and the respective fold change.

**Supplementary Table S1:** 

### The role of predictive miRNAs in regulating programmed cell death and MAP kinase

To gain insights into the biological processes involving the up- and down-regulated miRNAs, we performed two different enrichment analyses. First, we carried out a cutoff-free miRNA pathway enrichment analysis that takes the rank of each miRNA into account. Second, we performed a specific pathway enrichment analysis for the 9 significant miRNAs in comparison to all other remaining miRNAs. Details on both approaches are provided in the Materials section.

With respect to the first analysis, miRNAs were sorted in the descending order according to the levels of over-expression in patients developing cancer, *i.e.*, the first miRNA in the sorted list was the miRNA whose expression was most strongly increased. As robust criterion for the degree of up-regulation, we computed the area under the receiver operator characteristics (ROC) curve (AUC). We identified biochemical categories predominantly associated with miRNAs at the top of the sorted list. Of the more than 14,000 categories included in the miRNA Enrichment Analysis and Annotation tool (miEAA), 1966 pathways and gene ontologies were significantly associated with the development of cancer (*P* < 0.05; the rank-sum based method implemented in miEAA). miRNAs with the most significantly up-regulated expression (Table S2) were associated with regulation of MAP kinase activity, integrin signaling pathway, and focal adhesion (*P* = 2.3 × 10^−5^). miRNAs with the most down-regulated expression that were associated with these three pathways were hsa-miR-200b-3p and hsa-miR-199b-5p. The full list of significant pathways is provided in Table S2.

**Supplementary Table S2:** 

**Figure 3:**
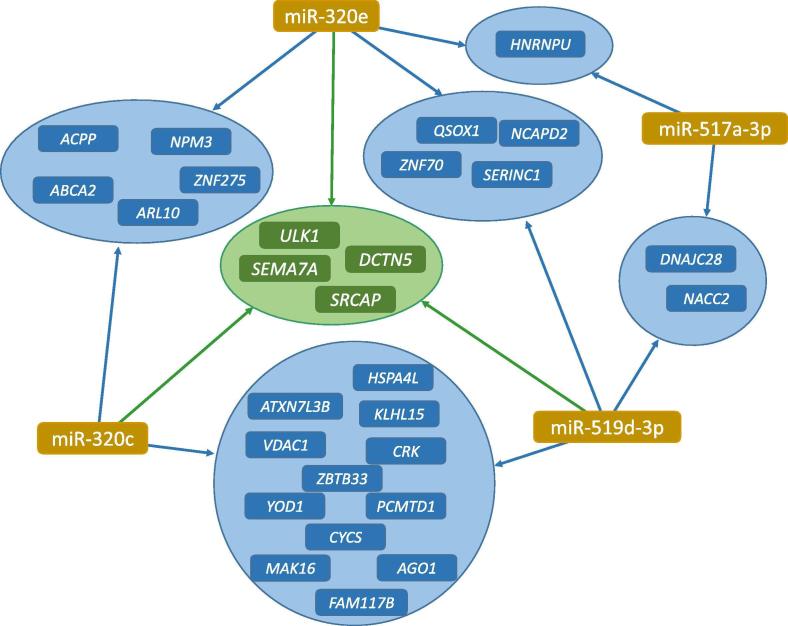
**miRNA-target interaction network for the four core miRNAs** The graphic shows for the four core miRNAs miR-320e, miR-517a-3p, miR-519d-3p, and miR-320c the joint target genes from the miRTarbase. miRNA nodes are colored in brown, genes targeted by two miRNAs are colored in blue and genes targeted by three miRNAs are colored in green.

Since the aforementioned approach does not necessarily focus on the most significant miRNAs but on the general relevance of miRNAs in the pathways considered, we specifically analyzed the biological categories of the 9 significant miRNAs detected in the previous section. Of these 9, 5 miRNAs were found in at least one significant pathway (*P* < 0.05; hypergeometric test as implemented in miEAA). In detail, miR-548d-5p, miR-320c, and miR-519d-3p are associated with the Gene Ontology (GO) “lipid binding”. miR-517a-3p, miR-320e, and miR-519d-3p are all located on chromosome 19 with miR-517a-3p and miR-519d-3p belonging to the same miRNA cluster and being co-located with a distance of around 10,000 bases. Both miR-320c and miR-519d-3p are involved in the GO category “programmed cell death”. Figure 3 provides a miRNA–target gene interaction network showing that four genes, namely *SRCAP*, *DCTN5*, *ULK1*, and *SEMA7A*, are targeted each by three of the nine miRNAs.

### Down-regulation of miR-150 in patients without cancer history

In addition to the information whether COPD patients developed cancer, anamnestic data on previous cancers had also been collected in COSYCONET (Figure 1). We performed an analysis of variance (ANOVA) with four groups of patients including (1) patients who had no cancer history nor developed cancer, (2) patients who had no cancer history but developed cancer, (3) patients with a cancer history who did not develop cancer, and (4) patients who had a history of cancer and developed cancer.

**Figure 4:**
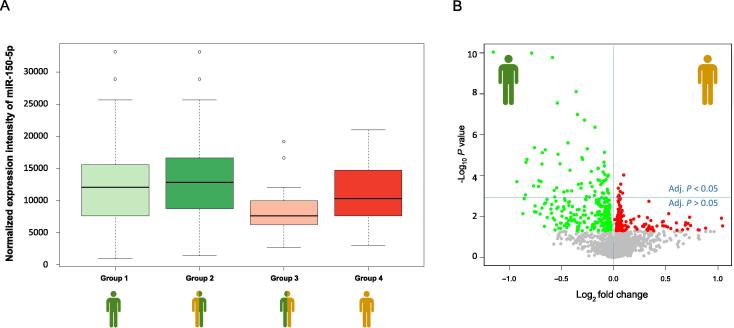
**Box-Whisker and Volcano plots for miRNAs including the information of a cancer history** **A.** Box whisker plot for miR-150-5p for the four patient groups shown in Figure 1. Patients in Group 3 showed significantly decreased expression of miR-150-5p. **B.** Volcano plot for the two extreme groups: Group 1 and Group 4. Green dots indicate miRNAs with higher expression in blood samples from Group 1, while red dots represent miRNAs with higher expression in blood samples from Group 4 according to the unadjusted *P* < 0.05. All dots with unadjusted *P* ≥ 0.05 are colored in gray. The horizontal blue line separates miRNAs that are significant following Benjamini–Hochberg adjustment (above the line; adjusted *P* < 0.05) and miRNAs that are not significant anymore following the adjustment (below the line).

We found a significant influence of cancer history on miRNA expression levels. The expression changes described above were frequently especially evident for patients without previous cancer history as exemplified using miR-150-5p (Figure 4A). Here, the down-regulation of miR-150-5p was most significant in patients without previous cancer history (Groups 3; *P* = 0.003; ANOVA).

This observation prompted us also to compare the “extreme” groups, *i.e.*, COPD patients that had no history of cancer and did not develop cancer (Group 1) with patients that had a history of cancer and developed cancer (Group 4). In this comparison, we observed a down-regulation in miRNA expression in cancer patients with *P* values which were, however, two to three orders of magnitude lower than those of the initial analysis as shown in Figure 4B. The two most significant miRNAs miR-409-3p and miR-133b had adjusted *P* values of 1.3 × 10^−7^. These analyses suggest that a cancer history can have a substantial impact on molecular miRNA profiles and should be included as confounding variable. Details for expression of each miRNA in both groups along with the fold change and the *P* values are presented in Table S3.

**Supplementary Table S3:** 

### miRNA signatures are predictive for detecting patients developing cancer without known cancer history

To check whether combinations of miRNAs have the ability to predict the development of cancer we next performed pair-wise classifications with gradient-boosted trees using small sets of 2–13 miRNAs as described in the Methods section. While the performance to predict a newly developing cancer in patients who had a history of cancer was not significantly better than random (accuracy of 50.5% using 8 miRNAs), it was possible to successfully predict newly developing cancers in patients without previous history of cancer. Since impact of the observed accuracy (71.5%) is limited by the unbalanced sample sizes, we also calculated the AUC value as a more informative performance measure. As a result, we obtained an AUC value of 74.7% using only two miRNAs, namely miR-450a-5p and miR-200b-3p, the latter being among the two most down-regulated miRNAs in the initial analysis. The best classification performance with an accuracy of 85.0% and an AUC of 87.4% was reached between patients who had a history of cancer and did not develop cancer (Group 2) and patients who have no history of cancer but developed cancer (Group 3) using three miRNAs (miR-450a-5p, miR-4677-3p, and miR-9-3p). The detailed classification results are presented in Table S4.

**Supplementary Table S4:** 

### Validation of the microarray data using qRT-PCR

**Figure 5:**
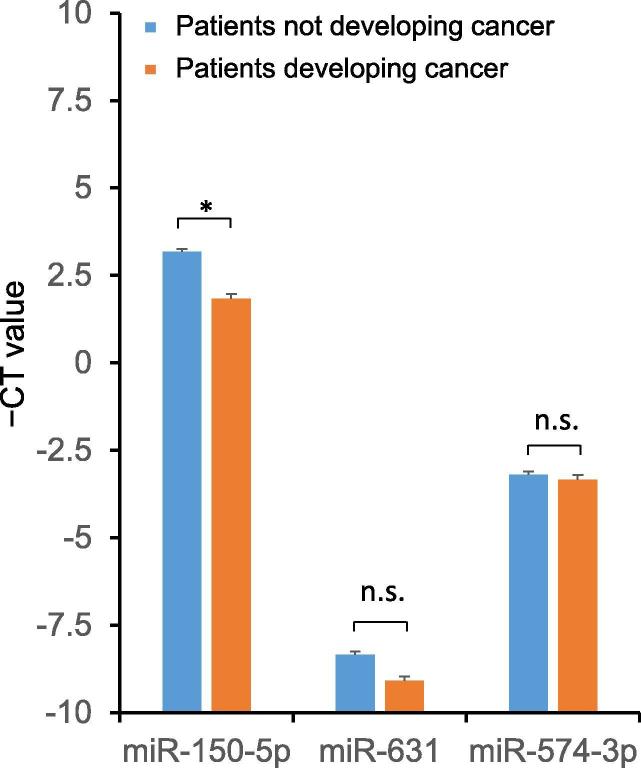
**qRT-PCR validation of the expression of three miRNAs** The graphic shows the CT values of expression in blood from patients developing cancer (n = 31) and those not developing cancer (n = 56) for the three miRNAs that were evaluated by qRT-PCR. * indicates significant difference in miRNA expression (*P* < 0.05; two-tailed *t*-test) between the two patient groups. n.s., non significant.

In order to validate the results obtained by microarray analysis, especially the predominant down-regulation of the miRNA expression, and to provide evidence that the down-regulation is not a general artifact or bias in the microarrays due to normalization, we performed qRT-PCR on a subset of samples including all samples of patients that developed a new tumor where enough material was left (31 of the original 33 cases) and 56 random controls that did not develop a tumor. First, we selected the aforementioned miR-150-5p, whose expression was down-regulated 1.47 fold in the original microarrays (see Table S1). In line with the microarray data, expression of miR-150-5p was significantly decreased by 2.52 fold in patients with future tumors compared to controls (*P* = 5.71 × 10^−7^). Besides this clearly down-regulated miRNA, we chose two candidates with lower fold changes (Table S1). The expression of miR-631 (fold change in the microarray analysis was 1.02) was 1.67 fold reduced in patients with future tumors as shown in PCR. Likewise, the expression of miR-574-3p that was 1.28 –fold down-regulated in the microarrays was slightly decreased by 1.1 fold as shown in PCR. Due to the smaller sample number in the validation, the down-regulation of the latter two miRNAs was however not significant in the qRT-PCR analysis. The RT-qPCR data are presented in Figure 5.

### The limited influence of age/gender/smoking history and other factors

We utilized clinical data of COSYCONET study to examine potential confounding factors. Smoking history and age are two risk factors that may have an influence on cancer and COPD [28], [29]. Although case and control cohort were similar with respect to these factors (see Table 1), we analyzed whether miRNAs correlated with age or individual smoking habits. None of the miRNAs was significantly linked to the number of years of smoking, nor to the number of cigarettes per day, nor to pack years (adjusted *P* > 0.05). There were, however, differences in miRNA expression between current and former smokers, most significantly for miR-92b-3p. Similarly, we found differences between patients who never smoked versus former or current smokers. Expression of 11 miRNAs was significantly correlated with age and the strongest correlations were found for miR-342-5p and miR-31-5p. Similarly, expression of three miRNAs was significantly associated with gender with most strong association found for miR-1912. These findings emphasize the need to match the groups with respect to these confounders. miRNAs affected by the confounders may either be excluded in building reliable statistical models for predicting newly developed cancers or be taken into account by using adjusted values for miRNA expression. All *P* values are provided in Table S5.

**Supplementary Table S5:** 

### miRNAs are correlated to other clinical factors relevant to lung function

We additionally analyzed the potential dependence of miRNA expression on other clinical information as coded in 84 parameters provided in the baseline description of COSYCONET [27]. ANOVA was used to test the dependence of miRNA expression on categorical features, whereas linear correlation coefficients were used on non-categorical features (*P* values are provided in Table S5). For most of the parameters, including the Global Initiative for Chronic Obstructive Lung Disease (GOLD) spirometric categories, no miRNA remained statistically significant after adjustment for multiple testing. However, we found that expression of 19 miRNAs, most strongly miR-4723-5p, was significantly correlated with the 6-min walk distance following adjustment for multiple testing. Additionally, expression of 3 miRNAs was found to be correlated with the long term oxygen therapy (LTOT) oxygen volume (l/min), comorbidity sarcoidosis, the diagnosis of atopic eczema, and chronic venous insufficiency, respectively. The respective miRNAs were usually different from those identified to be relevant for cancer development, and the effect sizes were moderate.

These results indicate that smoking status, age, gender, and comorbidities have an influence on miRNA expression. Nonetheless, the strongest associations were found for the development of cancer, especially in the absence of previous cancer.

## Discussion

In the present study, we analyzed blood samples collected in the COSYCONET COPD study. The large-scale collection of samples from patients without cancer at baseline, but with follow-up data, allowed to address the question of early diagnostic miRNA signatures for cancer. Limitations of the present study include the nature of the cancer diagnosis, which was patient-reported and the lack of information about the type of cancer besides lung cancer.

A common challenge in high-throughput molecular measurements is the low sampling density, *i.e.*, the fact that many more features (p) than individuals (n) are considered (p ≫ n challenge), which requires an adjustment of significance levels. One challenge with the present study is the imbalance between patients not developing cancer and those developing cancer. Since 33 cancer cases were available, one logical approach would have been to select 33 controls. However, given the above addressed p ≫ n challenge, this would likely not lead to reliable results. In the present study, 2549 human miRNAs were included compared to a number of 534 COPD patients. About 6% of patients developed cancer within 54 months, hence the groups were not balanced. Nonetheless, the effect sizes were strong enough and concordant to our power calculation such that 9 of 269 nominally significant markers miRNAs remained significant following adjustment for multiple testing. Furthermore, the current set-up with more non-cancer cases than cancer cases more likely corresponds to a realistic clinical setting than a mixture of 50% cases and 50% controls, especially if the signature will be applied to a screening set up, in which case numbers are usually one to two orders of magnitude below controls.

Our experimental methodology and distribution-based normalization techniques do not allow to identify global pattern alterations. The 9 most significant markers were all down-regulated. As shown by the volcano plots in Figure 2, Figure 4, there are also up-regulated miRNAs, suggesting that there is no overarching or global down-regulation pattern.

Since lung cancer is among the frequently investigated cancer entities, it is not surprising that a large fraction of the markers described in the present study has already been identified in other studies [30], [31], [32] but the heterogeneity of studies (different blood collection devices, measurement platforms, cohort characteristics) renders direct comparisons difficult. Considering the challenges described above, an analysis for specific cancer sub-types would not be feasible even with detailed histological data of the patients.

We applied two miRNA set enrichment analyses, one using the set of nine significant miRNAs (two tailed adjusted *P* value of the *t*-test) and one rank-based approach that considers the ranking of all miRNAs measured in the study without using hard cut-off values. As a result, we identified a network of 4 miRNAs including miR-517a-3p, miR-320e, miR-519d-3p, and miR-320c, and 28 target genes including the central target genes *SRCAP*, *DCTN5*, *ULK1*, and *SEMA.* The majority of these genes and miRNAs have previously been correlated to lung cancer. For example, a suppressed translation and degradation of ULK1 represents a potential mechanism for autophagy limitation in lung cancer [33], whereas miR-320c was described as core predictor for lung and other cancers [34].

Different experimental approaches have been proposed for miRNA classification [35]. In the present study, we experienced a high performance in building prediction models using gradient boosted trees combined with selecting features (miRNAs) according to their ANOVA *F*-values.

Altogether, we found an enriched fraction of markers that were less expressed in lung cancer with specific functional categories including MAP kinase, integrin signaling, and focal adhesion pathways. There were promising classification results using miRNA signatures for several pair-wise classification scenarios. To achieve more robust prediction, larger cohorts of patients developing cancer are however required. According to data from our miRNA tissue repository [36], the majority of down-regulated miRNAs was not specific for lung tissue, which is consistent with our previous data indicating a limited overlap between blood cell-based profiles (*e.g.*, from lymphocytes or red blood cells) with tissue-based profiles [37].

Using the prospective characteristics of the COSYCONET study, we observed molecular differences between the COPD patients later developing cancer and patients who did not develop cancer, indicating that miRNA signatures from blood have a potential to predict lung cancer in early stages, especially if patients had not suffered from cancer previously.

The analysis of a variety of clinical parameters revealed an influence of some of these parameters on miRNA profiles, which could affect their predictive power for lung cancer.

Although the application of miRNAs in clinical care still bears substantial challenges [25], there is a steadily increasing evidence supporting that miRNAs have a significant diagnostic potential. Our study suggests such a potential for early detection or prediction of lung cancer in COPD patients. In addition to the validation using other technologies, such as qRT-PCR presented in this study, a validation using an independent cohort is the logical next step to provide further evidence for the predictive power of the miRNA signatures discovered in this study.

## Materials and methods

### Study subjects and set-up

We used the baseline and follow-up datasets of the COSYCONET cohort study (visit 1), which is a prospective, multi-center, observational study in Germany [27]. Between 2010 and 2013, 2741 COPD patients were included. In addition to the baseline visit, follow-ups were scheduled at 6, 18, 36, and 54 months. At each visit, a large panel of assessments was performed, such as clinical history, smoking history, spirometry, body plethysmography, carbon monoxide (CO) diffusing capacity, PAXGene blood sampling, 6-min walk distance, electrocardiogram, and echocardiography. Patient-reported physician-based diagnoses of cancer of any kind were used to identify eligible individuals to be included in the study. Furthermore, a control cohort of COPD patients not developing cancer matched for age/sex/smoking history was included. The biosamples used (PAXGene blood samples) were taken from the baseline visit. The primary outcome measure was a positive cancer diagnosis of any kind during the follow-up in the COSYCONET study.

A sketch of the study design is presented in Figure 1. The follow-up time for the occurrence of cancer was 54 months, which is consistent with the study set up of COSYCONET. Such long follow-up periods are reasonable since previous analyses identified pre-cancerous miRNA signatures several years prior to the cancer diagnosis [38].

Out of all 2741 cases recorded by COSYCONET, we analyzed 33 COPD patients that developed cancer during the follow-up period. As for the numbers of controls, *i.e.*, COPD patients not developing cancer, the rational was as follows: selecting 33 controls with a power of 0.95 at an alpha error rate of 0.05 corresponds to an effect size of 0.9 for single miRNAs. Since previous studies show that miRNAs have lower effect sizes, we selected 500 controls yielding an effect size of 0.65 for single miRNAs. The control cohort was selected to keep potentially confounding variables similar between cases and controls (see Table 1).

Local ethics committee approved the study and all patients contributing to the study provided written informed consent.

### RNA isolation

RNA isolation was performed as described previously [39]. In short, RNA from whole blood samples collected in PAXgene Blood Tubes (BD Biosciences, Franklin Lakes, NJ) was isolated using PAXgene Blood miRNA Kit (Qiagen, Hilden, Germany) according to manufacturers’ instructions in a semi-automated fashion using Qiacube robot (Qiagen) to ensure reproducibility. RNA concentration was measured with Nanodrop spectral photometer (ThermoFisher Scientific, Waltham, MA) and RNA Integrity was assessed using RNA Nano Bioanalyzer Kit (Agilent Technologies, Santa Clara, CA).

### Microarray measurement

Considering the suggested substantial influence of library preparation on high-throughput sequencing studies [40], [41], we profiled the miRNA expression in blood samples using microarray analysis. miRNA expression measurements were performed as described previously by using the Agilent microarray system with the Human miRBase V21 microarrays [36], [42], containing 2549 mature human miRNAs. In short, RNA was dephosphorylated and then labeled with Cy3-pCp. Labeled RNA samples were hybridized to the arrays for 20 h at 55 °C. After washing and drying, arrays were scanned in an Agilent microarray scanner at 3-µm resolution in double-path mode. Raw expression values were extracted using Feature Extraction Software (Agilent Technologies, Santa Clara, CA). Altogether, we selected 580 patients from COSYCONET. For 534 samples, both, the RNA extraction and microarray measurement yielded high-quality results, while 46 samples failed quality control, *i.e.*, either RNA concentration or RNA integrity number (RIN) value is too low. These samples were excluded prior to further measurement or statistical analysis.

The microarray data are available upon request.

### Reverse transcription-quantitative PCR

RT-qPCR was performed using the miScript PCR system (Qiagen, Hilden, Germany). For a subset of 87 samples (31 with future tumor and 56 controls) we determined the expression levels of three miRNAs, *i.e.*, miR-150-5p, miR-574-3p, and miR-631. *RNU6B* and *SNORD48* were used as endogenous controls. In detail, 100 ng total RNA was reversely transcribed using miScript RT II kit, and 1 ng cDNA was used for qPCR using miScript SYBR Green reagent and respective miScript Primers (Qiagen) on a StepOnePlus cycler (Applied Biosystems, Foster City, CA). All reactions were set up in duplicates. Data were analyzed using StepOneSoftware (Applied Biosystems).

### Statistical analysis

All computations were performed using R (version 3.0.2) based on scripts developed for previous projects [39]. Following background subtraction and computation of the expression intensity of a miRNA by calculating the median of all replicates of that miRNA on each microarray, data were normalized using quantil normalization. To test whether miRNAs follow a normal or a log-normal distribution, Shapiro Wilk tests on the normalized and log-normalized data were computed. Since in both cases miRNAs that were not normally (or log-normally) distributed were discovered, we applied besides the parametric tests (for categorical features, two-tailed *t*-tests and ANOVA were performed) also non-parametric tests (here, the rank-based unpaired two-tailed Wilcoxon–Mann–Whitney test was applied). Both hypothesis tests, *t*-test and Wilcoxon–Mann–Whitney test, showed a high correlation to each other (correlation of 0.76, *P* < 10^−16^). In the manuscript, we focus on the more common *t*-test *P* values but because of the challenges described above and for the sake of completeness, all raw and adjusted *P* values computed by the *t*-test and the Wilcoxon–Mann–Whitney test are provided in Table S1). In case of multiple ties, *P* values were validated by using an exact implementation considering ties in EDISON-WMW [43].

For continuous features, Pearson correlation coefficients were computed. For the comparison of age, pack years, smoking status, and gender between the two cohorts, *t*-tests, contingence tables, and Fisher’s exact 2 × 2 test were used. To draw inferences on the involved biochemical pathways, the miEAA annotation [44], which is built on the well-known GeneTrail framework [45], was employed. As most other pathway analysis tools, miEAA and GeneTrail support not only a gene set-based analysis using, *e.g.*, Fisher’s test or the hypergeometric test, but also cutoff-free analyses. In the first case, the significant miRNAs could be compared to the non-significant miRNAs in our study and we would ask whether the significant miRNAs accumulate on one or several pathways. In this case, a miRNA with *P* = 0.049 would be considered while a miRNA with *P* = 0.051 (having basically the same information content) would belong to the background set. For this reason, we also implemented and applied cutoff-free analysis. The miRNAs are ranked by their *P* values and the sorted list of miRNAs is processed from top to bottom. Whenever a miRNA belonging to a pathway is found, a running sum is increased by (*m–l*) and otherwise the running sum is decreased by *l*. Here, *m* is the number of all miRNAs and *l* is the number of miRNAs on the current pathway. The *P* value for a pathway computes as the total number of all running sums exceeding the maximum value of the actual running sum. This *P* value is calculated by enumerating all potential distributions in an efficient manner relying on dynamic programming [46]. Using miEAA, over 14,000 pathways and other categories were tested sequentially using the two previously described approaches. Targeted gene–miRNA interactions were assessed using miRTargetLink [47].

If not mentioned explicitly, all *P* values were subjected to adjustment for multiple testing. The level of significance was assumed at *P* = 0.05 and significance values were adjusted for multiple testing if not mentioned explicitly.

### Machine learning

Predictions models were created based on the quantil normalized expression values of the miRNAs. All models were built using the gradient boosted tree implementation of the LightGBM framework. To account for the class imbalance, we weighted the classes according to their ratios. Hyperparameter tuning of the number of leaves, trees, and features was applied via grid search in a 5-time repeated 5-fold cross-validation. In this procedure only features with the highest ANOVA *F*-value were selected. All reported performance values refer to the cross-validation metrics. The final reported features were determined using the best performing model on the whole dataset.

## Authors’ contributions

AK initiated the study, performed biostatistical analyses, and drafted the manuscript. TF performed the classification analyses. NL, TL, and CD contributed to the measurement of microarray profiles. MK contributed to the statistical analyses. CB worked on the miRNA Set Enrichment Analysis using miEAA. CV, FB, and RAJ contributed to the COSYCONET study. CH selected the patients from the COSYCONET study and provided clinical annotations. HL co-initiated the study and contributed to the pathway analysis. EM co-initiated the study and drafted the manuscript. RB contributed to the set-up of this study and the COSYCONET study, provided clinical information and drafted the manuscript. All authors read and approved the final manuscript

## Competing interests

MK and TL are employed by Hummingbird Diagnostics GmbH.

## References

[1] Decramer M., Janssens W. (2013). Chronic obstructive pulmonary disease and comorbidities. Lancet Respir Med.

[2] Ho C.H., Chen Y.C., Wang J.J., Liao K.M. (2017). Incidence and relative risk for developing cancer among patients with COPD: a nationwide cohort study in Taiwan. BMJ Open.

[3] Durham A.L., Adcock I.M. (2015). The relationship between COPD and lung cancer. Lung Cancer.

[4] Zakharkina T., Heinzel E., Koczulla R.A., Greulich T., Rentz K., Pauling J.K. (2013). Analysis of the airway microbiota of healthy individuals and patients with chronic obstructive pulmonary disease by T-RFLP and clone sequencing. PLoS One.

[5] Sekine Y., Katsura H., Koh E., Hiroshima K., Fujisawa T. (2012). Early detection of COPD is important for lung cancer surveillance. Eur Respir J.

[6] Raviv S., Hawkins K.A., DeCamp M.M., Kalhan R. (2011). Lung cancer in chronic obstructive pulmonary disease: enhancing surgical options and outcomes. Am J Respir Crit Care Med.

[7] Biswas A., Mehta H.J., Folch E.E. (2018). Chronic obstructive pulmonary disease and lung cancer: inter-relationships. Curr Opin Pulm Med.

[8] Cho W.C., Kwan C.K., Yau S., So P.P., Poon P.C., Au J.S. (2011). The role of inflammation in the pathogenesis of lung cancer. Expert Opin Ther Targets.

[9] Kornum J.B., Svaerke C., Thomsen R.W., Lange P., Sorensen H.T. (2012). Chronic obstructive pulmonary disease and cancer risk: a Danish nationwide cohort study. Respir Med.

[10] Buist A.S., Vollmer W.M., McBurnie M.A. (2008). Worldwide burden of COPD in high- and low-income countries. Part I. The burden of obstructive lung disease (BOLD) initiative. Int J Tuberc Lung Dis.

[11] Chen W., Brehm J.M., Manichaikul A., Cho M.H., Boutaoui N., Yan Q. (2015). A genome-wide association study of chronic obstructive pulmonary disease in Hispanics. Ann Am Thorac Soc.

[12] Cho M.H., Castaldi P.J., Wan E.S., Siedlinski M., Hersh C.P., Demeo D.L. (2012). A genome-wide association study of COPD identifies a susceptibility locus on chromosome 19q13. Hum Mol Genet.

[13] Wain L.V., Shrine N., Artigas M.S., Erzurumluoglu A.M., Noyvert B., Bossini-Castillo L. (2017). Genome-wide association analyses for lung function and chronic obstructive pulmonary disease identify new loci and potential druggable targets. Nat Genet.

[14] Kowalczyk M.S., Higgs D.R., Gingeras T.R. (2012). Molecular biology: RNA discrimination. Nature.

[15] Jankowsky E., Harris M.E. (2015). Specificity and nonspecificity in RNA–protein interactions. Nat Rev Mol Cell Biol.

[16] Dang X., Qu X., Wang W., Liao C., Li Y., Zhang X. (2017). Bioinformatic analysis of microRNA and mRNA Regulation in peripheral blood mononuclear cells of patients with chronic obstructive pulmonary disease. Respir Res.

[17] Kara M., Kirkil G., Kalemci S. (2016). Differential expression of microRNAs in chronic obstructive pulmonary disease. Adv Clin Exp Med.

[18] Inamura K., Ishikawa Y. (2016). MicroRNA in lung cancer: novel biomarkers and potential tools for treatment. J Clin Med.

[19] Song Y., Yu X., Zang Z., Zhao G. (2018). Circulating or tissue microRNAs and extracellular vesicles as potential lung cancer biomarkers: a systematic review. Int J Biol Markers.

[20] Leidinger P., Brefort T., Backes C., Krapp M., Galata V., Beier M. (2016). High-throughput qRT-PCR validation of blood microRNAs in non-small cell lung cancer. Oncotarget.

[21] Keller A., Leidinger P., Bauer A., Elsharawy A., Haas J., Backes C. (2011). Toward the blood-borne miRNome of human diseases. Nat Methods.

[22] Wei J., Li F., Yang J., Liu X., Cho W.C. (2015). MicroRNAs as regulators of airborne pollution-induced lung inflammation and carcinogenesis. Arch Toxicol.

[23] Keller A., Leidinger P., Vogel B., Backes C., ElSharawy A., Galata V. (2014). miRNAs can be generally associated with human pathologies as exemplified for miR-144. BMC Med.

[24] Leidinger P., Keller A., Borries A., Huwer H., Rohling M., Huebers J. (2011). Specific peripheral miRNA profiles for distinguishing lung cancer from COPD. Lung Cancer.

[25] Backes C., Meese E., Keller A. (2016). Specific miRNA disease biomarkers in blood, serum and plasma: challenges and prospects. Mol Diagn Ther.

[26] Cho W.C. (2011). Promises and challenges in developing miRNA as a molecular diagnostic tool for lung cancer. Expert Rev Mol Diagn.

[27] Karch A., Vogelmeier C., Welte T., Bals R., Kauczor H.U., Biederer J. (2016). The German COPD cohort COSYCONET: aims, methods and descriptive analysis of the study population at baseline. Respir Med.

[28] Mannino D.M., Buist A.S. (2007). Global burden of COPD: risk factors, prevalence, and future trends. Lancet.

[29] Molina J.R., Yang P., Cassivi S.D., Schild S.E., Adjei A.A. (2008). Non-small cell lung cancer: epidemiology, risk factors, treatment, and survivorship. Mayo Clin Proc.

[30] Fan L., Qi H., Teng J., Su B., Chen H., Wang C. (2016). Identification of serum miRNAs by nano-quantum dots microarray as diagnostic biomarkers for early detection of non-small cell lung cancer. Tumour Biol.

[31] Jin X., Chen Y., Chen H., Fei S., Chen D., Cai X. (2017). Evaluation of tumor-derived exosomal miRNA as potential diagnostic biomarkers for early-stage non-small cell lung cancer using next-generation sequencing. Clin Cancer Res.

[32] Keller A., Leidinger P., Borries A., Wendschlag A., Wucherpfennig F., Scheffler M. (2009). miRNAs in lung cancer – studying complex fingerprints in patient's blood cells by microarray experiments. BMC Cancer.

[33] Allavena G., Boyd C., Oo K.S., Maellaro E., Zhivotovsky B., Kaminskyy V.O. (2016). Suppressed translation and ULK1 degradation as potential mechanisms of autophagy limitation under prolonged starvation. Autophagy.

[34] Pal J.K., Ray S.S., Pal S.K. (2016). Identifying relevant group of miRNAs in cancer using fuzzy mutual information. Med Biol Eng Comput.

[35] Saha S., Mitra S., Yadav R.K. (2017). A stack-based ensemble framework for detecting cancer microRNA biomarkers. Genomics Proteomics Bioinformatics.

[36] Ludwig N., Leidinger P., Becker K., Backes C., Fehlmann T., Pallasch C. (2016). Distribution of miRNA expression across human tissues. Nucleic Acids Res.

[37] Fehlmann T., Ludwig N., Backes C., Meese E., Keller A. (2016). Distribution of microRNA biomarker candidates in solid tissues and body fluids. RNA Biol.

[38] Keller A., Leidinger P., Gislefoss R., Haugen A., Langseth H., Staehler P. (2011). Stable serum miRNA profiles as potential tool for non-invasive lung cancer diagnosis. RNA Biol.

[39] Keller A., Backes C., Haas J., Leidinger P., Maetzler W., Deuschle C. (2016). Validating Alzheimer's disease micro RNAs using next-generation sequencing. Alzheimers Dement.

[40] Backes C., Sedaghat-Hamedani F., Frese K., Hart M., Ludwig N., Meder B. (2016). Bias in high-throughput analysis of miRNAs and implications for biomarker studies. Anal Chem.

[41] Fehlmann T., Reinheimer S., Geng C., Su X., Drmanac S., Alexeev A. (2016). cPAS-based sequencing on the BGISEQ-500 to explore small non-coding RNAs. Clin Epigenetics.

[42] Ludwig N., Becker M., Schumann T., Speer T., Fehlmann T., Keller A. (2017). Bias in recent miRBase annotations potentially associated with RNA quality issues. Sci Rep.

[43] Marx A., Backes C., Meese E., Lenhof H.P., Keller A. (2016). EDISON-WMW: exact dynamic programing solution of the Wilcoxon–Mann–Whitney test. Genomics Proteomics Bioinformatics.

[44] Backes C., Khaleeq Q.T., Meese E., Keller A. (2016). miEAA: microRNA enrichment analysis and annotation. Nucleic Acids Res.

[45] Backes C., Keller A., Kuentzer J., Kneissl B., Comtesse N., Elnakady Y.A. (2007). GeneTrail–advanced gene set enrichment analysis. Nucleic Acids Res.

[46] Keller A., Backes C., Lenhof H.P. (2007). Computation of significance scores of unweighted Gene Set Enrichment Analyses. BMC Bioinformatics.

[47] Zafari S., Backes C., Leidinger P., Meese E., Keller A. (2015). Regulatory microRNA networks: complex patterns of target pathways for disease-related and housekeeping microRNAs. Genomics Proteomics Bioinformatics.

